# Massive non-incisional abdominal wall hernia caused by abdominal wall weakness resulting from childhood radiation therapy: a case report

**DOI:** 10.1080/23320885.2022.2059485

**Published:** 2022-04-27

**Authors:** Tatsuya Ichida, Yuki Otsuki, Koichi Ueda

**Affiliations:** a Department of Plastic Surgery, Ueyama Hospital, Neyagawa, Osaka, Japan; b Department of Plastic and Reconstruction Surgery, Osaka Medical and Pharmaceutical University, Osaka, Japan

**Keywords:** Abdominal wall hernia, non-incisional, non-traumatic, radiation therapy

## Abstract

Huge abdominal wall hernias after radiation therapy in the absence of any previous surgical incisions or trauma are rare and, to the best of our knowledge, have not previously been reported. we report a patient with a massive hernia caused by abdominal wall weakness resulting from childhood radiation therapy.

## Introduction

Classification of abdominal wall hernias is controversial; however, they can generally be categorized on the basis of cause as congenital, acquired, incisional, or traumatic [1]. Acquired abdominal wall hernias are caused by repetitive stress on weak anatomical points in the abdominal wall, examples including umbilical, epigastric, lumber (both superior and inferior), and Spigelian hernias [1,2]. In contrast, because they are caused by weakness of the abdominal wall resulting from surgical incisions or trauma, incisional and traumatic hernias can occur in any region of that wall. This article reports a case of a massive, non-traumatic, non-incisional abdominal wall hernia caused by abdominal wall weakness resulting from childhood radiation therapy.

## Case report

**Figure 1.**
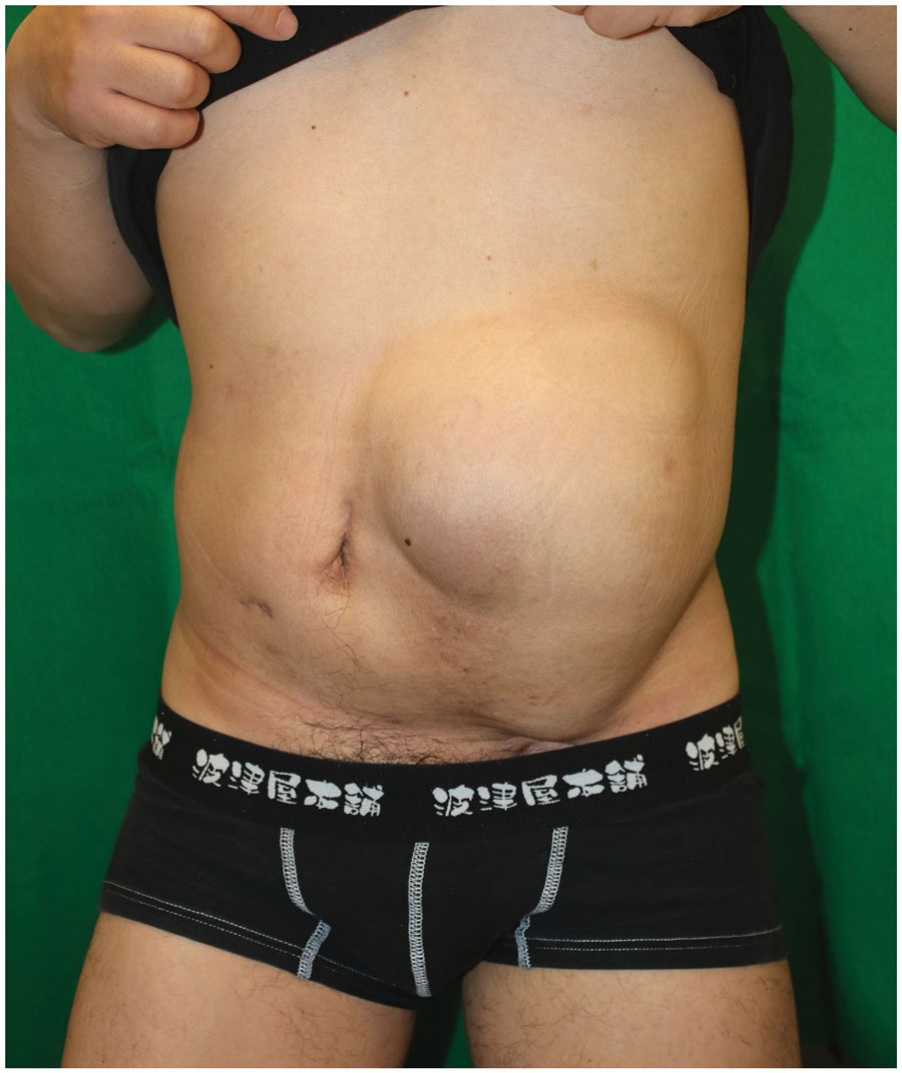
Preoperative appearance. A 43-year-old man presented a left abdominal wall hernia and bulge.

**Figure 2.**
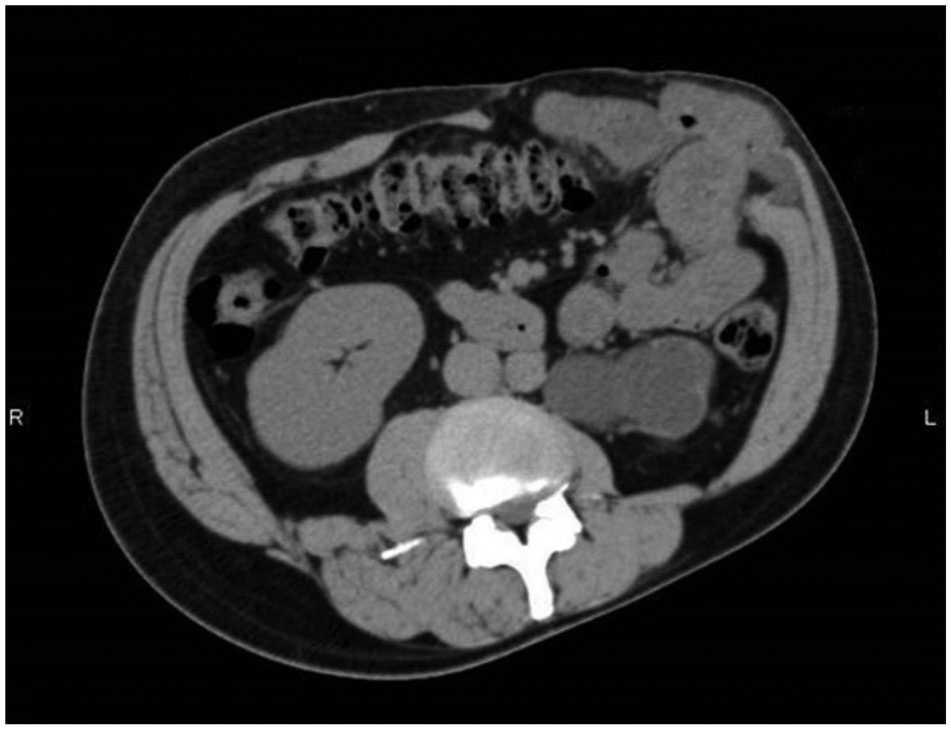
Axial CT image of the abdomen. It shows abdominal wall defect and the herniated small bowel and colon.

A 43-year-old man was referred to our hospital with an abdominal wall hernia that had developed at the age of 42. Physical examination on the initial visit showed a huge bulge over almost all of the left half of the abdomen (Figure 1). There were no surgical or traumatic scars on the abdominal wall. A CT scan of the abdomen and pelvis showed an abdominal wall defect, herniated small bowel and colon (Figure 2), and atrophy of the erector spinae and rectus abdominis muscles and subcutaneous tissue. Moreover, unilateral pelvic atrophy and scoliosis of the lumbar spine were detected. He had a history of childhood testicular cancer that had been treated by unilateral orchiectomy and radiation therapy to regional lymph nodes in the abdomen and pelvis and had undergone bone elongation for impaired left femoral bone growth at the age of 8. Since then, he has had no relevant symptoms or evidence of recurrence. However, he had undergone ureterolithotripsy at the age of 42 and presented about one week later with massive abdominal distention. In the absence of any surgical or traumatic scar in the abdominal wall, abdominal wall weakness resulting from radiation therapy to abdominal and pelvic lymph nodes was suspected. Details of radiation range and dose were unavailable. However, this hypothesis was supported by the presence of pigmentation and thinness of the skin over the area of abdominal distention. The hernial orifice was so large that strangulation was extremely unlikely to occur; thus, a decision was made to perform surgery at the patient's convenience. Six months after his initial visit to our hospital, small intestinal perforation occurred, requiring emergency surgical repair. Eight months after that, definitive hernia repair was performed under general anesthesia. The skin was so thin that the abdominal cavity was reached immediately. There were minor adhesions between the abdominal wall and intestinal tract and laparotomy was performed without any problems. The hernial orifice was 21 × 14 cm and there were focal ascites in the lower abdomen. Examination of the entire small intestine revealed white erosions in the serosa of about 80 cm of the jejunum. Although this possibly represented radiation-induced enteritis, no obvious intestinal damage was detected, so a wait-and-see approach was adopted. After washing the intraperitoneal cavity, the defect was closed, the cranial end with a linear closure and the remaining 17 × 8 cm defect with fascia lata.

**Figure 3.**
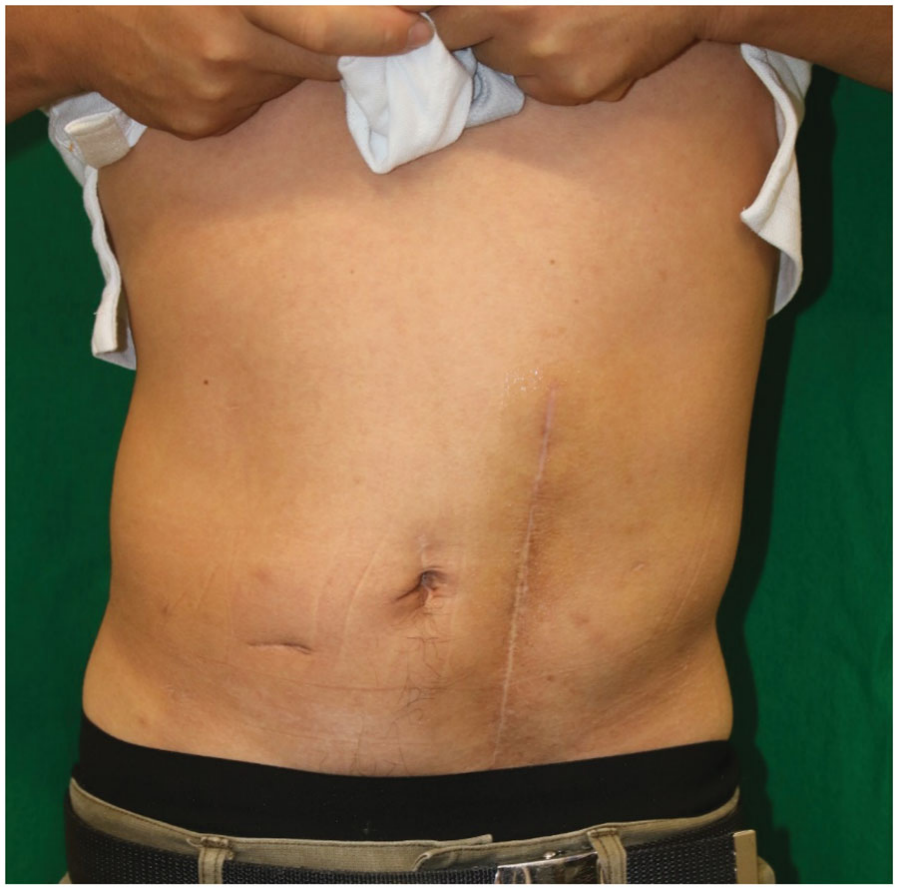
Postoperative appearance 6 months after last surgery. There was no sign of recurrence of the hernia and bulge.

**Figure 4.**
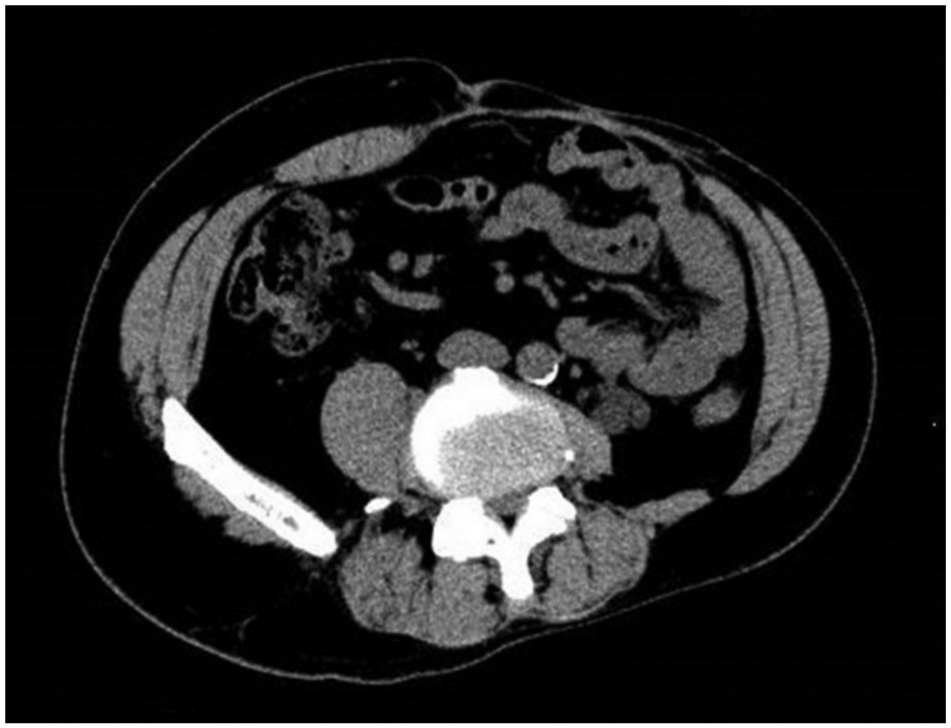
Axial CT image of the abdomen 13 months after last surgery. There is no sign of recurrence of the hernia.

About one year later, a hernia recurred at the site of the linear closure. The 4 × 3 cm hernial orifice was managed by direct closure. There was no clinical or CT evidence of further hernial recurrence at a 13-month follow-up visit (Figures 3 and 4).

## Discussion

Abdominal wall hernias can generally be categorized on the basis of cause as congenital, acquired, incisional, or traumatic [1]. Acquired abdominal wall hernias occur in weak anatomical sites and can be categorized by their general location as midline, median, or paramedian. Midline hernias include epigastric and umbilical hernias, median hernias include supravesical hernias, and paramedian hernias include Spigelian and interparietal hernias [1]. In contrast, incisional and traumatic hernias can occur in any region of the abdominal wall because they are caused by weakness resulting from surgical incisions or trauma.

We here report a non-traumatic, non-incisional abdominal wall hernia that did not occur in a weak anatomical site. However, consistent with the patient having undergone radiation therapy to the abdominal and pelvic area, the skin overlying the hernia was darkly pigmented and extremely thin. Moreover, a CT scan had shown unilateral pelvic atrophy, scoliosis of the lumbar spine and atrophy of the erector spinae and rectus abdominis muscles, and subcutaneous tissue. Radiation therapy can cause impairment of bone growth [3] and soft-tissue hypoplasia. The characteristic response to radiation-induced injury varies according to tissue type, with atrophy predominating in epithelial tissue whereas fibrosis predominates in stromal tissue [4]. Atrophy of the epithelial tissue and fibrosis of the stromal tissue may have resulted in thinning and loss of elasticity of the abdominal wall, which in turn may have resulted in the fragility of the abdominal wall. We, therefore, attributed our patient’s scoliosis and hemiatrophy of the pelvis to radiation-induced asymmetrical growth plate impairment and growth disturbances, as previously described [5]. In their report on soft tissue hypoplasia long after radiation therapy for Wilms tumor, Paulino et al. cited rates of radiation-related muscular hypoplasia and scoliosis of 16.7% and 42.9%, respectively [5]. These findings indicate that radiation therapy in childhood can affect the surrounding tissue, resulting in darkly pigmented, thin skin and impairment of soft tissue and bone growth. Our patient’s abdominal wall weakness was thus likely attributable to childhood radiotherapy and the main factor responsible for his huge abdominal hernia in the absence of any surgical scar.

There are many options for treating abdominal hernias, including suture repair, mesh repair, autologous fascia grafting, and the components separation technique. The optimal procedure is selected on the basis of the size and location of the hernial orifice, condition of the soft tissue, and other factors. Primary suturing may be appropriate when the hernia is small. However, the rate of recurrence after this procedure is reportedly 43% [6]; thus, another surgical procedure may be necessary to minimize recurrence. When the hernial orifice is large, insertion of some material is needed to close it, the surgical mesh being the most widely used. Mesh repair may be a treatment option for this case, but it may not be the best option. A partial list of mesh-related complications includes infection requiring mesh removal, mesh mechanical failure, mesh bulging, chronic pain, chronic inflammatory reaction, and mesh erosion into abdominal viscera [7]. The guidelines for laparoscopic treatment of ventral and incisional state that in many giant incisional hernias with a horizontal defect of more than 10 cm, standard open techniques and the laparoscopic intraperitoneal onlay mesh repair are insufficient [7].

The components separation technique, which was first described in 1990 [8], is a technique for abdominal wall reconstruction in patients with large midline hernias that cannot be closed primarily. It is useful in the presence of contamination, which is a contraindication to the use of prosthetic material. However, the subsequent re-herniation rate is relatively high (32%) [9]. It has long been recognized that surgery on tissues that have been subjected to moderate to high doses of radiation months to years earlier is associated with an increased risk of complications, including infection, delayed healing, wound dehiscence, fistula formation, and wound necrosis [10]. Hence, in these cases, artificial materials should be avoided, autologous tissue being preferable. The most widely used material is fascia lata because it can provide large patches. If the skin and soft tissue defects are too large for use of fascia lata, an anterolateral femoral or tensor fascia lata flap can be used to reconstruct the skin.

In our patient, the skin at the hernial site was only pigmented and did not require resection. Because the hernial orifice was large, fascia lata was used to close it. Disa et al. reported using autologous fascia lata in 32 patients with deficient abdominal walls in whom prosthetic material was contraindicated; their hernia recurrence rate was 9% [11]. They selected fascia patches because of their potentially large size, ease of procurement, and limited donor site morbidity.

It is important to be aware that late-onset abdominal wall hernias can occur in individuals who have undergone abdominal irradiation many years earlier, even in the absence of previous surgery or trauma. The clinical course of this patient is currently good, but only a little over a year has passed, and long-term follow-up is desired in the future.

## Statement of informed consent

Informed consent was obtained from the patient of the study.
